# Evaluating the Consent Preferences of UK Research Volunteers for Genetic and Clinical Studies

**DOI:** 10.1371/journal.pone.0118027

**Published:** 2015-03-11

**Authors:** Susan E. Kelly, Timothy D. Spector, Lynn F. Cherkas, Barbara Prainsack, Juliette M. Harris

**Affiliations:** 1 Centre for the Study of Life Sciences (Egenis), University of Exeter, Exeter, United Kingdom; 2 Department of Twin Research & Genetic Epidemiology, King’s College London, London, United Kingdom; 3 Department of Social Science, Health & Medicine, King’s College London, London, United Kingdom; Cardiff University, UNITED KINGDOM

## Abstract

**Objectives:**

To establish the views of research volunteers on the consent process; to explore their views on the consent process in different research scenarios; to inform debate on emerging models of consent for participation in research.

**Design, Setting and Participants:**

2,308 adult volunteers from the TwinsUK Registry (www.twinsuk.ac.uk) completed an online survey about their views on the consent process for use of their DNA and medical information in research. Their views on the re-consenting process in different scenarios were assessed.

**Results:**

The majority of volunteers preferred to be informed of the identity of the main researcher of a study in which they are participating, which is contrary to current practice. Over 80% were willing to complete the consent process online instead of face to face. On the whole, respondents did not view their DNA differently from their medical information with regard to the consent process. Research participants were more willing to give broad consent to cover future research if their DNA was to be used by the original researcher than by another researcher, even if the disease under investigation varied, in contrast to the traditional ‘gold standard’ whereby specific consent is required for all new research projects.

**Discussion:**

In some scenarios, research participants reported that they would be comfortable with not signing a new consent form for future research uses of their data and DNA, and are comfortable with secure, online consent processes rather than traditional face-to-face consent processes. Our findings indicate that the perceived relationship between research participants and researchers plays an important role in shaping preferences regarding the consent process and suggest that this relationship is not captured by traditional consent processes. We argue that the development of new formats of consent should be informed by empirical research on volunteers’ perceptions and preferences regarding the consent process.

## Introduction

Biomedical research is often underpinned by large international and collaborative datasets. It increasingly entails the integration of different types of data, including DNA, other molecular data, and clinical information. Examples of this modality of research include Genomics England, the initiative recently announced by the UK government to create a database of 100,000 anonymised whole genome sequences matched with clinical data, and the UK Biobank resource of 500,000 volunteers with genetic and clinical data. Accompanying these developments have been debates in ethics, legal and research communities about appropriate models of informed consent and participant protection [1–7].

Contemporary biomedical research drawing upon ‘big’ digital datasets reconfigures the challenges pertaining to consent in at least three ways: First, unlike in the paper age where data could be locked away or irrevocably destroyed, digital data can be much more easily copied, transferred, restored, and potentially also accessed by unauthorised parties. Second, the use of data-mining methods has spurred the production of unsolicited or ‘incidental findings’, i.e. findings that are not pertinent to the initial research question. This has given rise to a wide debate about what ‘incidental findings’ from individual datasets should be fed back to research participants, and by whom [8, 9, 10, 11]. Third, the increasing number and range of data stored and processed have intensified debates about who should decide on access and use of these datasets [12, 13]. Taken together, these debates have challenged the assumption that consent can be fully informed. It is impossible to predict all future scenarios of data use and the risks that these may pose [14, 15, 16, 17].

Traditionally, research consent involving DNA and medical information has taken place face-to-face, and the gold standard has been for consent to be given for samples and information to be used in a specific research protocol during a specific time frame. Increasingly, these measures are being identified as either impractical or even as impediments to research [18]. As a result, new models of consent are being developed, such as ‘portable consent’. Portable consent is an experimental bioethics protocol for the protection of research participants who donate data for unspecified research purposes [19]. De-identified data can then be accessed by any researcher who agrees to protect the research participants and their data under the terms of the consent, and to ensure the accessibility of published derivative results. Moreover, there are a number of initiatives for using information technologies for obtaining and ‘updating’ consent on an on-going basis, such as the ‘dynamic consent’ model pioneered by the EnCoRe project. The aim of the project is to develop and assess a system of electronic communication that would allow patients and donors to monitor how their tissue sample and personal information are being used and make decisions about how they can be used in the future [1,20]. Another innovation employing information technologies is Edward Dove and colleagues’ wiki-governance model [21]. Such models aim at allowing greater flexibility for research participants, who could pick and choose what parts of a research project they opt into and which ones they opt out from (and change their minds over time), instead of giving a binary ‘yes’ or ‘no’ answer in a one-off interaction at the beginning of a project. Other approaches argue in favour of decreasing bureaucracy around consent and propose to improve and expand formats for the compensation of actual harms instead [17].

We strongly believe that new instruments and processes around consent should be informed by the views and preferences of actual research volunteers. We thus set out to explore, in a large cohort of research volunteers in the UK, understandings and preferences on issues raised in various emerging models of consent for participation in research. We were particularly interested in assessing issues related to the degree and nature of control respondents wished to retain over their medical information and their DNA; participants’ preferences about how informed consent should be organized; and their views on ‘ownership’ of their samples and clinical information. We also assessed whether participants had different preferences regarding how consent and ownership should be organized for use of their DNA compared to their (non-genetic) clinical information. There is significant ethical scholarship on these points, but little empirical research. Exceptions [22, 23, 24, 25, 26, 27, 28] mostly have been qualitative in nature and therefore smaller in scope (for exceptions see [29, 30, 31, 32]). To complement these studies on this topic, we sought to establish the views of members of a large volunteer research registry on the consent process and to explore the degree and nature of control volunteers would prefer over their DNA and medical information in different research scenarios.

## Methods

### Institutional background and research setting

In Spring 2013, we conducted a survey of research volunteers enlisted in the TwinsUK registry [33, 34]. TwinsUK was set up in 1992 with members being recruited through national media campaigns and from other smaller twin registers. The initial aim of the registry was to investigate the incidence of osteoporosis, osteoarthritis and other rheumatologic diseases in unaffected women aged 40 and over, who were recruited as healthy volunteers. Since then the registry has expanded; however there are still more females than males due to the original aim of the research.

The focus of the research has greatly expanded in recent years and twins are invited to take part in a range of studies investigating the genetic and environmental aetiology of age related complex traits and diseases. Currently, the majority of twins who join the registry learn about it via word of mouth, through the web-site and from TwinsUK media appearances. Hence, volunteers are not selected for any particular trait. Any twin pair in the UK over the age of 18 can become a member of TwinsUK. Members of the TwinsUK registry are comparable to age-matched general population singletons for a broad variety of medical and behavioural traits [35]. The volunteers are predominantly white. They represent all educational and socio-economic levels and come from all regions of the United Kingdom.

On joining the registry, volunteers are invited to complete online or postal health questionnaires and to come to St. Thomas’s Hospital for a clinical study visit and to donate blood for DNA and biochemical markers. Research volunteers sign a generic paper consent where they agree to take part in ethics-approved research and for their data to be shared with approved researchers. The study was approved by the St Thomas’ Hospital Research Ethics Committee, now known as the NRES Committee London Westminster.

The Department of Twin Research, King’s College London hosts a Twin Volunteer Advisory Panel which was established in 2009. Its goal is to ensure that the opinions and views of volunteers are taken onboard during the development and running of research programmes at the Department. One of the roles of the Volunteer Advisory Panel is piloting research questionnaires. Further information about the Twin Volunteer Advisory Panel can be found here: http://www.twinsuk.ac.uk/twin-zone/twin-volunteer-advisory-panel/.

### Our study

The survey was designed to ascertain volunteers’ views on practical aspects of the consent process for medical research. The specific topic areas were identified from existing debates in literatures on new understandings and processes of consent. The survey was not preceded by qualitative work by this team addressing these issues, although this is further work we would like to undertake. Questions and responses were drafted initially by the authors, and then amended following piloting by the volunteer advisory panel, and members of the Department of Twin Research’s questionnaire development team as well as other staff members.

Questions were included about participants’ willingness to share medical information and/or DNA with researchers in different situations, particularly including research into mental health, criminal behaviour, and intelligence. These are commonly cited controversial areas of research, and thus are a good way to determine whether participants’ views on the consent process change depending on the context of research.

The survey included a definition of informed consent: ‘A process by which a subject voluntarily confirms his or her willingness to participate in research, after having been informed of all relevant aspects of the research.’ An example scenario was also provided, in which participants were told to imagine they were a volunteer in a UK-based medical research study (approved by an ethics committee) investigating the causes of a serious disease which involved the donation of blood for DNA and gathering of clinical information. Most of the questions resulted in the participant selecting the appropriate pre-coded and fixed responses. The full text of the questionnaire is printed in S1 Dataset.

The questionnaire was emailed to all volunteers who participate currently in TwinsUK online questionnaires (4284 in total). The questionnaire was hosted on a private server at the Department of Twin Research which required the volunteers to enter their TwinsUK unique identifier and unique password before completing the questionnaire. The server sits inside a firewall protected network. The 4284 respondents are aged between 18 and 87 with a mean age of 53, and are 88% female and 12% male.

### Statistical Analysis and Data Sharing

The statistical software STATA 10 was used to analyse the data [36]. Tests of significance (binomial test and Pearson’s chi-squared) were used as appropriate, and regression analysis was used to identify any significant associations with gender and age. Our main focus was to identify the responses of people actively engaged in the research process; the data were not weighted to reflect the characteristics of the adult population of the UK.

The Department of Twin Research is committed to providing the scientific community with unrestricted access to the phenotype data from the TwinsUk cohort (www.twins.ac.uk/data-access/open-access/).

The full set of responses is available in S1 Dataset.

## Results

Of the 4284 subjects invited to participate, 2308 completed the online consent questionnaire (54% response rate), with a mean age of 55, age range = 18–87 years (89% female, 11% male).

A survey of a random sample of 35 non-responders was also performed after the online survey had closed. (We targeted non-responders specifically with a new survey request 10 months after the conclusion of the first survey.) The mean age of these previous non-responders (who now completed the survey) was similar to the main study (53 versus 55) and there were no significant differences between the responses of these 35 previous non-responders and the initial 2308 responders to any of the questions in the survey.

### Consent process

When respondents were asked if they thought the consent form should state the name and contact details of the main researcher running the study, a significant proportion of respondents (73%) gave a positive response, with increasing age significantly associated with this response (p<0.01). When those who had answered affirmatively were subsequently asked why they felt this was necessary, four in five (81%) endorsed statements that this would enable them to trust the researcher to act in a responsible manner and would allow them to contact the researcher if necessary. Again, older respondents were significantly more likely to endorse this (p<0.01). Those who responded ‘no’ to the original question were not asked for reasons for their choice. (Please refer to the questionnaire in S1 Dataset for the full response set.)

One in five respondents (19%) were unwilling to provide online consent via a secure website, with women and older respondents significantly more likely to prefer the traditional consent model with a paper form completed with the researcher present (p<0.05).

### Re-consenting for New Studies

The initial questions in the study were asked in relation to the consent process for the use of medical information and DNA for ‘Disease A’ (which was not specified further than being serious). Participants in the survey were then presented with future potential research scenarios in relation to the consent process for the use of their medical information and DNA. We will first present results pertaining to DNA only. If further studies on the initial disease (Disease “A”) were to be carried out by the **same initial** researcher, a significant majority of respondents (58%) were happy to give permission for their DNA to be used without being re-contacted. This proportion dropped to 44% if their DNA were to be used by the same researcher to study a **different** disease. Asked whether they would want to be re-consented if a **different investigator** was to use their DNA to conduct research on either the same or a different disease, respondents were only half as likely to give their consent without being re-contacted (31% and 26% respectively). Three in five respondents expressed a preference to sign a new consent form if the additional study were to be carried out by a different investigator (61% when the study involves the initial disease, and 62% when the study relates to a different disease) (see Table 1). Increasing age was significantly associated with the willingness of respondents for their DNA to be used in all these scenarios without needing to be re-contacted (p<0.05).

**Table 1:** Results for question: ‘Would you give your permission for your DNA to be used in…’.

	Yes, WITHOUT being re-contacted	Yes, but only after signing NEW consent form	No, not willing
a. A NEW research study on ‘Disease A’ by the SAME researcher	**58**	**40**	**1**
b. A NEW research study on ‘Disease A’ by a DIFFERENT researcher	**31**	**61**	**8**
c. A NEW research study on a DIFFERENT disease by the SAME researcher	**44**	**53**	**4**
d. A NEW research study on a DIFFERENT disease by a DIFFERENT researcher	**26**	**62**	**12**

Figures are in percentages.

Of those respondents who would want to sign a new consent form, or not participate at all, in the scenario of a different researcher and different disease (62%), 65% strongly agreed with the statement ‘I want to know who will have access to my DNA before I decide’. 54% strongly agreed with the statement that ‘[i]t is important to me to have control over what happens to my DNA’, and with the statement ‘I want to know about what the future research might involve before I decide’. About half (48%) strongly endorsed the statement expressing concern about privacy if other researchers have access to their DNA. Only 30% strongly agreed with the statement ‘I may change my mind about participating in future research’, with younger respondents significantly more likely to agree with this statement (p<0.05).

A very similar pattern of responses was found when the same series of questions about re-consenting was repeated with respect to medical information instead of DNA (see Table 2).

**Table 2:** Results for question: ‘Would you give permission for your MEDICAL INFORMATION to be used in…’

	e. Yes, WITHOUT being re-contacted	Yes, but only after signing NEW consent form	No, not willing
f. A NEW research study on ‘Disease A’ by the SAME researcher	**61**	**37**	**1**
g. A NEW research study on ‘Disease A’ by a DIFFERENT researcher	**31**	**63**	**6**
h. A NEW research study on a DIFFERENT disease by the SAME researcher	**44**	**53**	**3**
A NEW research study on a DIFFERENT disease by a DIFFERENT researcher	**26**	**63**	**11**

Figures are in percentages.

Nearly all (over 90%) of those respondents who were willing for their DNA to be used in a new study on a different disease by a different researcher without being re-contacted first seemed to view it as a natural extension of the consent they had already given. They most prominently endorsed the statements ‘I am happy to help all medical research,’ and ‘I would like to save the researchers money and time.’

When asked about potential further use of their medical information and DNA to be used in ethics-approved research (based in a European, Asian, or North American university), at least half the respondents (50%-57%) would prefer to sign a new consent form in each case, with women significantly more likely to request re-consent than men (p<0.05). The remaining respondents were equally likely to be unwilling for their data to be used at all as to give permission for their data to be used without being recontacted (20–30%). When asked about use of their medical information and DNA by a UK-based pharmaceutical company, a similar proportion (57%) would prefer to sign a new consent form, however more than a third (37%) were willing for their data to be used without re-consent with increasing leniency with age (p<0.01). Only 6% were unwilling for their data to be used at all.

When the focus of our consent questions related to different types of research—behavioural research into mental health, criminal behaviour and intelligence—respondents were still mostly willing for their DNA and medical information to be used. However, over half (55–57%) would want to be re-consented for the different scenarios (see Table 3), with younger respondents more likely to request re-consent in all cases (p<0.01). For all questions pertaining to re-consenting, we plan to probe deeper into the reasons underpinning these answers in a further qualitative study.

**Table 3:** Results for question: ‘Would you give your permission for your MEDICAL INFORMATION AND DNA from the “study of Disease A” to be used in the UK for ….’.

	Yes, WITHOUT being re-contacted	Yes, but only after signing NEW consent form	No, not willing
a. Research into mental health	**39**	**57**	**3**
b. Research into criminal behaviour	**35**	**55**	**9**
c. Research into intelligence	**38**	**56**	**5**

Figures are in percentages.

When participants in the study were asked whether they would be happy to be automatically enrolled in an approved study when receiving treatment in an NHS hospital, they were significantly more likely to agree if the study was related to the illness for which they were being treated, compared with a study unrelated to their illness (46% and 23% respectively p<0.01; see Table 4). There was a significant increased willingness to enroll with age in both scenarios (p<0.01), however men were significantly more likely than women to enroll if the study was unrelated to their illness (p<0.05). We did not specify whether such ‘automatic enrollment’ would involve an opt-out option.

**Table 4:** Results for questions: ‘If you were in an NHS hospital receiving treatment for an illness, would you be happy to be AUTOMATICALLY enrolled ….’

…in any approved research RELATED to this illness?		
No 28	Yes 46	Undecided 26
… in any approved research NOT related to this illness?		
No 45	Yes 23	Undecided 33

Figures are percentages.

There was no clear consensus concerning participating in a ‘research commons’—i.e. a system whereby volunteers can render their anonymised data and information accessible to a pool of approved researchers without giving specific consent for each research project (see Question 12 on the questionnaire for exact wording). Whilst 43% indicated they would be likely to ‘upload their genomic and health data onto a secure internet site’ nearly one in three (31%) were undecided and the remaining quarter (26%) said they were unlikely to do so. There was a significant age effect with men significantly more likely than women to express willingness to enroll in a research commons (p<0.01).

### Ownership of DNA and medical information

There was a strong sense among our volunteers that they retain some kind of ownership of their medical information and DNA when it is being used in research. We did not provide a definition of the term ‘ownership’ as we were interested in how volunteers would respond intuitively. As many as three in four respondents (75%) agreed with the statement ‘My medical information and DNA belong to me, and I have allowed the researcher to use them for this study.’ There was a significant trend with age; younger people were more likely to believe they retained ownership of their DNA and medical information (p<0.05). One in five respondents (21%) felt that these data and information belonged equally to themselves and the researchers; whilst only 3% said that the researcher now had exclusive ownership. This is despite the fact that in enlisting in the TwinsUK registry, they had given permission for their samples and medical information to ‘be shared with the NIHR Bioresource and *bone fide* researchers and stored for future research studies aimed at identifying interactions between genes, the environment and disease’ in every consent form they had previously signed.

## Discussion

The ascendency of ‘big data’ science in medical research is accompanied by re-examination of traditional models of informed consent for participation in research. Traditionally it has been argued that research participants need to be informed about any intended research for which their data or sample will be used [17]. Under this model, a research subject should be re-contacted for consent for new research uses of their data; yet, this model is increasingly being challenged as an impediment to research and unworkable in large scale international biobanking forms of research. So far, little empirical research has explored the actual understandings and preferences of research participants. An exception is a recent study exploring the preferences of close to 4,700 adults in the US, which resonates with our findings and argues that support for broad consent is contingent on sufficient information being provided about data use [29]. Our empirical findings from a large volunteer research population thus contribute much needed information on research participants’ preferences on consent and related issues.

The importance of a relationship with a known researcher (which could be read as ‘trust’) emerged as a strong theme shaping consent preferences. Our findings suggest that in some circumstances many research participants are comfortable with not signing a new consent form for future research uses of their data and DNA. This is a challenge to the ‘gold standard’ of research consent and suggests that a more flexible approach could be adopted. Quite clearly, the vast majority of volunteers in this study expressed loyalty and trust towards their initial research investigator and articulated more reservations when new avenues of research or new investigators become involved. Our results showed that age and gender may influence the need to be re-consented, with older respondents and men more likely to not request re-consenting in some scenarios.

Trust has figured prominently in ethical discussions regarding biobanks, largely as a theoretical concept, rather than empirical finding as in our study. If the values and goals pursued by the biobank or research organisation, and their financial stakes, can be incorporated into the consent form, then it may not be necessary to re-consent participants each and every time new research applications arise, with the notion that it would save researchers time and money possibly being a key message [17, 37, 38, 39].

Our findings further suggest that secure, online consent processes are acceptable to a large proportion of research participants. The fact that ours was an online survey, however, may account for it having been answered primarily by participants who are more comfortable with web-based applications. Further research would be needed to establish the generalisability of our results in this respect.

Consent procedures should express a form of ‘partnership’ such as articulated in the House of Lords Science and Technology Committee report Human Genetic Databases: Challenges and Opportunities [40, 41]. Lunshof and colleagues have also argued that if the partnership between research institutions and research participants is taken seriously, greater reciprocity in the rights to data on both sides is needed [12]. The quest for a more genuine ‘partnership’ between the participant and the researcher in the sense of greater veracity and reciprocity, together with the finding that such relationships are key with regard to research participation, should provide the basis for further development of the consent process. Perhaps such a growing emphasis on genuine partnership will also foster understandings of shared ownership of data, in contrast to our finding that many respondents felt they retained ownership of their DNA and medical information.

It was interesting that over half the responders would be willing for their DNA and medical information to be used for research into behavioural, non-medical conditions such as criminal behaviour and intelligence; this is one of the areas we would want to explore in more depth in a follow-on qualitative study as it runs counter to some previous research.

### Limitations

We acknowledge that there are limitations to this study, including that closed question surveys are limited in not allowing the exploration of reasoning behind responses given. The survey was UK based and so interpretation of findings may be limited to that population. Volunteers have already decided to participate in research which may colour their views. Further, for historical reasons, the TwinsUK Registry has considerably more female than male members and has less ethnic diversity than the general population of the UK. However, it is known that women are more likely to volunteer for research than men. Future research needs to understand the preferences for research participation of people from more ethnically and culturally diverse backgrounds and engage them in the research process (but see [42]).

## Conclusion

Taken together, our findings suggest that new understandings and models of consent should reflect the preferences of research participants. We support Klaus Hoeyer’s insistence that there is great diversity between different kinds of biobanks and within preferences of donors and participants, and solutions need to be tailored to every biobank specifically, according to its purpose, context, and the characteristics and preferences of their participants [21]. At the same time, it is clearly apparent that the traditional model of consent needs re-evaluation. One of its main shortcomings is its overreliance on expectations of individual autonomy that assumes the potential research subject to be an isolated rational actor.

Our findings suggest that participants’ preferences regarding informed consent are deeply relational. This means that, as pointed out above, the trust they have in a particular researcher or research institution with whom they have developed a relationship is an important factor in the kind of consent they prefer. This relational aspect is underconceptualised in the literature (for an important exception see O’Neill’s work on trust [43]). We will also explore this finding and interpretation in further qualitative research. It has significant implications for emerging models of consent.

Future models of informed consent could allow for the accountability of researchers and at the same time, flexibility of use of data and materials. This would mean potentially that researchers fully take onboard the accountability relationships involved in research, a recognition that has long pertained (if it is not always abided by) in qualitative research communities.

The more we understand how participants form expectations about the handling of their information and materials, and the control they want to exert, the better. A better understanding of participants’ values and preferences, in turn, will enable biobanks and research organisations to better accommodate these. As a result, trust in the biobank or research organization is likely to increase as well.

This study has emphasised the importance of gaining the perspectives and understandings of actual research participants and future qualitative research will explore this in more depth.

Earlier work [44] on research participation in radiation experiments found that potential research participants had a great deal of trust in the judgments of their physicians in decisions about research participation. The authors concluded that responsible agents had an obligation to ensure that such trust was not misplaced. We interpret our findings in a complementary fashion—preferences about consent need to be understood in a relational way that can entail participants wanting someone else (such as a trusted researcher) to make decisions on their behalf.

## Supporting Information

S1 Dataset (DOCX)
