# Supplementary material for: Evaluating the Consent Preferences of UK Research Volunteers for Genetic and Clinical Studies
Source: PLoS One. 2015 Mar 11;10(3):e0118027. doi: 10.1371/journal.pone.0118027 (PMC4356519; doi:10.1371/journal.pone.0118027)
Supplement: S1 Dataset — (DOCX) [file pone.0118027.s001.docx]

**2308 RESPONSES, 11% MALE AND 89% FEMALE**

**DEFINITION:**

Informed consent for research can be defined as **‘A PROCESS BY WHICH A SUBJECT VOLUNTARILY CONFIRMS HIS OR HER WILLINGNESS TO PARTICIPATE IN RESEARCH, AFTER HAVING BEEN INFORMED OF ALL RELEVANT ASPECTS OF THE RESEARCH.’**

**EXAMPLE SITUATION:** Please imagine that you are a volunteer in a UK-based medical research study investigating the causes of a serious disease (which we will refer to as Disease A). The study has been approved by an ethics committee and includes both patients and healthy volunteers. As part of the study you are asked to complete a questionnaire about your current health and medical history (referred to throughout as ‘medical information’). You are also asked to donate blood for the analysis of your DNA. **YOU AGREE TO BE IN THE STUDY AND ARE GIVEN A CONSENT FORM TO SIGN.**

***SECTION A: THE CONSENT PROCESS***

Q1) Consent forms always include the name of the HOSPITAL or UNIVERSITY conducting the study. Do you think the consent form should ALSO state the name and contact details of the MAIN RESEARCHER running the study? (0) 􀁕 No – Go to Q3a **27%** (1) 􀁕 Yes – Go to Q2 **73%**

Q2) Here is a list of reasons why you might think the consent form should also state the name and contact details of the MAIN researcher running the study (and not just the hospital/University). On a scale of 1 to 5, where 1= strongly agree and 5 = strongly disagree, please indicate how much you agree or disagree with these reasons.

|  | Strongly agree  (1) | Tend to agree  (2) | Neither agree nor disagree(3) | Tend to disagree  (4) | Strongly disagree(5) |
| --- | --- | --- | --- | --- | --- |
| 1. I would trust the research project more if I knew exactly who was running it | **39%** | **41%** | **16%** | **3%** | **2%** |
| 1. I would feel that the main researcher would act more responsibly if their name and contact details are known by research volunteers | **45%** | **36%** | **13%** | **4%** | **3%** |
| 1. I would like to be able to contact them if I have questions about the research | **41%** | **40%** | **13%** | **4%** | **2%** |
| 1. I would like to look up information about the main researcher | **36%** | **39%** | **19%** | **5%** | **1%** |

Q3a) Consent usually involves face-to-face communication with a researcher and the signing of a paper form**.** As a volunteer in the ‘study of Disease A’ (as described in the introduction), would you be willing to receive information about the study and complete the consent process **ON-LINE USING A SECURE WEB-SITE INSTEAD OF FACE-TO-FACE?**

(0) 􀁕 No - Go to Q4 **19%** (1) 􀁕 Yes - Go to Q3b **81%**

Q3b) How would you PREFER to receive study information and give consent for the ‘study of Disease A’? (1) 􀁕 On-line (using a secure web-site) **50%**

(2) 􀁕 With a researcher physically present using a paper consent **9%**

(3) 􀁕 No preference **41%**

Q4) Having taken part in the ‘study of Disease A’, and provided researchers with your medical information and DNA, which of the following responses best describes how you feel towards your MEDICAL INFORMATION AND DNA?

(1) 􀁕 My medical information and DNA belong to ME, and I have allowed the researcher to use them for this study **75%**

(2) 􀁕 My medical information and DNA belong EQUALLY to me and to the researcher **21%**

(3) 􀁕 My medical information and DNA NO LONGER belong to me, but now BELONG to the researcher**3%**

(4) 􀁕 Don't know **2%**

***SECTION B: USE OF YOUR MEDICAL INFORMATION***

Having taken part in the ‘study of Disease A’, we are now interested in finding out how you feel about your **MEDICAL INFORMATION** from the ‘study of Disease A’ being used in a **NEW** UK-based research study (approved by an ethics committee). Your medical information will be anonymised and only the original researchers would be able to identify you.

**Q5) WOULD YOU GIVE YOUR PERMISSION FOR YOUR MEDICAL INFORMATION TO BE USED IN….(see a-d)**

|  | Yes, WITHOUT being re-contacted  (1) | Yes, but only after signing NEW consent form  (2) | No, not willing  (3) |
| --- | --- | --- | --- |
| 1. A NEW research study on ‘Disease A’ by the SAME researcher | **61%** | **37%** | **1%** |
| 1. A NEW research study on ‘Disease A’ by a DIFFERENT researcher | **31%** | **63%** | **6%** |
| 1. A NEW research study on a DIFFERENT disease by the SAME researcher | **44%** | **53%** | **3%** |
| 1. A NEW research study on a DIFFERENT disease by a DIFFERENT researcher | **26%** | **63%** | **11%** |

***SECTION C: USE OF YOUR DNA***

Having taken part in the study of ‘Disease A’, we are now interested in finding out how you feel about your **DNA** from the study of ‘Disease A’ being used in a **NEW** UK-based research study (approved by an ethics committee). Your DNA will be anonymised and only the original researchers would be able to identify you. (Please note that the previous similar question was about your medical information – THIS QUESTION IS ABOUT YOUR DNA)

**Q6) WOULD YOU GIVE YOUR PERMISSION FOR YOUR DNA TO BE USED IN….(see a-d)**

|  | Yes, WITHOUT being re-contacted  (1) | Yes, but only after signing NEW consent form  (2) | No, not willing  (3) |
| --- | --- | --- | --- |
| 1. A NEW research study on ‘Disease A’ by the SAME researcher | **58%** | **40%** | **1%** |
| 1. A NEW research study on ‘Disease A’ by a DIFFERENT researcher | **31%** | **61%** | **8%** |
| 1. A NEW research study on a DIFFERENT disease by the SAME researcher | **44%** | **53%** | **4%** |
| 1. A NEW research study on a DIFFERENT disease by a DIFFERENT researcher | **26% Go to Q7** | **62% Go to Q8** | **12% to Q8** |

Q7) Here is a list of reasons why you might be willing for your DNA to be used in A NEW research study on a DIFFERENT disease (i.e. not ‘Disease A’) by a DIFFERENT researcher –WITHOUT BEING RE-CONTACTED FIRST. On a scale of 1 to 5, where 1= strongly agree and 5 = strongly disagree, please indicate how much you agree or disagree with these reasons.

|  | **Strongly agree**  **(1)** | **Trend to agree**  **(2)** | Neither agree nor disagree(3) | **Tend to disagree**  **(4)** | Strongly disagree(5) |
| --- | --- | --- | --- | --- | --- |
| 1. I am happy to help all medical research | **85%** | **15%** | **1%** | **<1%** | **<1%** |
| 1. I see this as a natural extension of the consent I have already given | **75%** | **21%** | **2%** | **<1%** | **<1%** |
| 1. I trust the judgement of the researchers | **70%** | **24%** | **6%** | **<1%** | **<1%** |
| 1. I trust that ethics committees are acting in my best interest | **73%** | **23%** | **3%** | **<1%** | **<1%** |
| 1. I would like to save the researchers money and time | **79%** | **18%** | **3%** | **<1%** | **<1%** |

- **NOW GO TO Q9**

Q8) Here is a list of reasons why you may NOT be willing for your DNA to be automatically used in A NEW research study on a DIFFERENT disease (i.e. not ‘Disease A’) by a DIFFERENT researcher (i.e. you would rather not take part in the new study at all, or you would like to sign a new consent form). On a scale of 1 to 5, where 1= strongly agree and 5 = strongly disagree, please indicate how much you agree or disagree with these reasons.

|  | **Strongly agree**  **(1)** | **Tend to agree**  **(2)** | Neither agree nor disagree(3) | **Tend to disagree**  **(4)** | Strongly disagree(5) |
| --- | --- | --- | --- | --- | --- |
| 1. It is important for me to have control over what happens to my DNA | **54%** | **33%** | **9%** | **2%** | **<1%** |
| 1. I want to know about what the future research might involve before I decide | **54%** | **38%** | **5%** | **2%** | **<1%** |
| 1. I want to know who will have access to my DNA before I decide | **65%** | **28%** | **5%** | **1%** | **<1%** |
| 1. I may change my mind about participating in future research | **30%** | **33%** | **23%** | **11%** | **3%** |
| 1. I am concerned about my privacy if other researchers have access to my DNA | **48%** | **31%** | **14%** | **5%** | **1%** |

***SECTION D: FUTHER USE OF YOUR MEDICAL INFORMATION AND DNA***

Having taken part in the ‘study of Disease A’, we are now interested in finding out how you would feel about your MEDICAL INFORMATION and DNA collected during the ‘study of Disease A’ being used in other situations. All research is approved by an ethics committee, and all your information will be anonymised and only the original researchers would be able to identify you.

Q9) Would you give your permission for your MEDICAL INFORMATION AND DNA

to be used in a study DEVELOPING NEW DRUGS OR TREATMENTS for ‘Disease A’ at….(see a-d)

|  | Yes, WITHOUT being re-contacted  (1) | Yes, but only after signing NEW consent form  (2) | No, not willing  (3) |
| --- | --- | --- | --- |
| 1. a regulated UK-based pharmaceutical company | **37%** | **57%** | **6%** |
| 1. a University in Europe | **24%** | **57%** | **20%** |
| 1. a University in Asia | **20%** | **50%** | **30%** |
| 1. a University in North America | **20%** | **54%** | **25%** |
|  |  |  |  |

Q10) Would you give your permission for your MEDICAL INFORMATION AND DNA from the

‘study of Disease A’ to be used in the UK for ….(see a-c)

|  | Yes, WITHOUT being re-contacted  (1) | Yes, but only after signing NEW consent form  (2) | No, not willing  (3) |
| --- | --- | --- | --- |
| 1. Research into mental health | **39%** | **57%** | **3%** |
| 1. Research into criminal behaviour | **35%** | **55%** | **9%** |
| 1. Research into intelligence | **38%** | **56%** | **5%** |

Q11a). If you were in an NHS hospital receiving treatment for an illness, would you be happy to be AUTOMATICALLY enrolled in any approved research RELATED to this illness?

(0) 􀁕 No **28%** (1) 􀁕 Yes **46%** (2) 􀁕 Undecided **26%**

11b) If you were in an NHS hospital receiving treatment for an illness, would you be happy to be AUTOMATICALLY enrolled in any approved research NOT related to this illness?

(0) 􀁕 No **45%** (1) 􀁕 Yes **23%** (2) 􀁕 Undecided **33%**

Q12). Scientists in the USA have developed a new system whereby volunteers can upload their genomic (DNA profile) and health information WITHOUT identifiers (i.e. name, date of birth, etc) onto a secure Internet site. Approved scientists from around the world can then access this information for scientific research. If this were available in the UK, how likely would you be to upload your genomic and health information?

(1) 􀁕 Very likely **14%** (2) 􀁕 Fairly likely **29%** (3) 􀁕 Undecided **31%**

(4) 􀁕 Fairly unlikely **14%** (5) 􀁕 Very unlikely **12%**

Q14). Finally, in general, would you say your health is……?

(1) 􀁕 Excellent **18%** (2) 􀁕 Very good **43%** (3) 􀁕 Good **29%** (4) 􀁕 Fair **8%** (5) 􀁕 Poor **1%**
